# 3D printed mesh reinforcements enhance the mechanical properties of electrospun scaffolds

**DOI:** 10.1186/s40824-019-0171-0

**Published:** 2019-11-29

**Authors:** Nicholas W. Pensa, Andrew S. Curry, Paul P. Bonvallet, Nathan F. Bellis, Kayla M. Rettig, Michael S. Reddy, Alan W. Eberhardt, Susan L. Bellis

**Affiliations:** 1 Department of Biomedical Engineering, University of Alabama at Birmingham, Birmingham, USA; 2 Department of Cell, Developmental and Integrative Biology, University of Alabama at Birmingham, Birmingham, USA; 3 School of Dentistry, University of California at San Francisco, San Francisco, USA

**Keywords:** Electrospun scaffolds, 3D printing, Polycaprolactone, Mechanical properties, Tissue regeneration

## Abstract

**Background:**

There is substantial interest in electrospun scaffolds as substrates for tissue regeneration and repair due to their fibrous, extracellular matrix-like composition with interconnected porosity, cost-effective production, and scalability. However, a common limitation of these scaffolds is their inherently low mechanical strength and stiffness, restricting their use in some clinical applications. In this study we developed a novel technique for 3D printing a mesh reinforcement on electrospun scaffolds to improve their mechanical properties.

**Methods:**

A poly (lactic acid) (PLA) mesh was 3D-printed directly onto electrospun scaffolds composed of a 40:60 ratio of poly(ε-caprolactone) (PCL) to gelatin, respectively. PLA grids were printed onto the electrospun scaffolds with either a 6 mm or 8 mm distance between the struts. Scanning electron microscopy was utilized to determine if the 3D printing process affected the archtitecture of the electrospun scaffold. Tensile testing was used to ascertain mechanical properties (strength, modulus, failure stress, ductility) of both unmodified and reinforced electrospun scaffolds. An in vivo bone graft model was used to assess biocompatibility. Specifically, reinforced scaffolds were used as a membrane cover for bone graft particles implanted into rat calvarial defects, and implant sites were examined histologically.

**Results:**

We determined that the tensile strength and elastic modulus were markedly increased, and ductility reduced, by the addition of the PLA meshes to the electrospun scaffolds. Furthermore, the scaffolds maintained their matrix-like structure after being reinforced with the 3D printed PLA. There was no indication at the graft/tissue interface that the reinforced electrospun scaffolds elicited an immune or foreign body response upon implantation into rat cranial defects.

**Conclusion:**

3D-printed mesh reinforcements offer a new tool for enhancing the mechanical strength of electrospun scaffolds while preserving the advantageous extracellular matrix-like architecture. The modification of electrospun scaffolds with 3D-printed reinforcements is expected to expand the range of clinical applications for which electrospun materials may be suitable.

## Introduction

Substantial research is currently focused on the potential utility of electrospun scaffolds for clinical applications including the repair of diaphragm [1], bladder [2–4], and ligaments [5–8], as well as grafting procedures for bone [5, 9], skin [10–12], and vascular [13, 14] tissue. Electrospun scaffolds provide a 3-dimensional fibrous matrix with interconnecting pores, a feature that mimics the native extracellular matrix (ECM). Electrospun scaffolds are also highly tunable with regard to pore size and fiber degradation characteristics, and can be readily scaled up for commercial production. Furthermore, their high surface to volume ratio provides a suitable topography for cell adhesion and locomotion. To improve biocompatibility, methods have been developed to incorporate endogenous ECM molecules and/or growth factors to promote cell differentiation, survival, and/or proliferation [15]. However, one of the major disadvantages of electrospun scaffolds is that they have relatively poor mechanical properties (low strength and stiffness, high ductility) compared to many of the tissues they are designed to heal [16–18]. This limits their use in applications that require a material with relatively high mechanical strength and stiffness, such as bone or tendon repair.

Electrospun scaffolds are often produced as composite materials that blend ECM molecules such as collagen I with synthetic polymers which have higher tensile strength. Composite scaffolds integrate the favorable biochemical characteristics of native matrix molecules with the advantageous mechanical properties of synthetic polymers. One prevalent synthetic polymer used in electrospinning is poly(ε-caprolactone) (PCL). PCL is FDA-approved, biodegradable, and protocols for electrospinning PCL are well-established. Furthermore, numerous in vivo studies support the biocompatibility of PCL [19–21]. Other synthetic polymers used in electrospinning include poly (3-hydroxybutyrate-co-3-hydroxyvalerate) (PHBV) [22], poly (vinylidene fluoride) (PVD) [23], and poly (lactic acid) (PLA) [24]. While composite materials (e.g., PCL/collagen mixtures) are typically stronger than scaffolds composed solely of natural matrix molecules, the tensile strength of these composites rarely approaches the mechanical strength of tissues such as bone or tendon [8, 12]. For many regenerative therapies, it is thought that the mechanical properties of an implanted scaffold should match that of the target tissue [25]. Accordingly, new approaches are needed to enhance the mechanical properties of electrospun scaffolds to make them more suitable for applications in musculoskeletal repair and regeneration.

In the current study, we describe a novel method that utilizes both electrospinning and 3D printing to create reinforced electrospun scaffolds with improved mechanical properties. Specifically, scaffolds were electrospun using a combination of gelatin, a collagen-derivative that supports cell adhesion, and PCL, which provides mechanical support. A PLA mesh was then 3D printed onto one side of the electrospun material. The end result is a scaffold that retains the biocompatibility and favorable architecture of electrospun substrates, while having the enhanced mechanical strength imparted by the PLA mesh. Mechanical testing of the scaffolds revealed that the addition of the 3D printed mesh increased tensile strength by ~ 13 fold. Furthermore, the reinforced scaffolds had increased stiffness and reduced ductility. Finally, the reinforced scaffolds did not elicit any immune or foreign body response upon implantation into rat cranial defects. These results point to a promising new approach for improving the mechanical properties of electrospun scaffolds while preserving the beneficial characteristics of the electrospun layer.

## Materials and methods

### Scaffold synthesis

Scaffolds were prepared using hexafluoropropanol (HFP, Sigma-Aldrich, St. Louis, MO) to dissolve a 40:60 ratio by weight of PCL (10 kD, Scientific Polymer Products, Ontario, NY) to gelatin (Sigma-Aldrich, St. Louis, MO), respectively. Solutions of 40 wt% PCL (0.012 g) and 60 wt% gelatin (0.016 g) were created by adding HFP (2 mL) such that the solid weight of the mixture was 7.5% (PCL/gelatin) of the total solution weight. The 40:60 ratio was selected because it offers a suitable blend between the cell adhesion-promoting features of gelatin and the more favorable tensile properties of PCL. The PCL/gelatin/HFP solution was incubated for 2 h at 37 °C and then sonicated at 40 kHz (Branson 1510, Danbury, CT) for 20 min to ensure completely solubilization. The solution was taken up into a 3 mL syringe fitted with a blunt tip 27-gauge needle and passed through the needle using a syringe pump (Harvard Apparatus, Cambridge, MA) at a rate of 2 mL/hr. Voltage (20 kV) was applied to the syringe with a voltage supply (Gamma High Voltage Research, Ormond Beach, FL) to generate the electrospun fibers, and fibers were deposited onto a 20 rpm rotating collector plate. Once the electrospinning was complete, the scaffolds were placed in a desiccator for 24 h to remove any residual HFP solvent.

To create the reinforced mesh, scaffolds were placed in a 3D printer (Replicator 2.0, MakerBot, New York, NY), and the PLA network was deposited onto the scaffold at 185 °C (1.75 mm PLA filaments were purchased from MakerBot). Two types of meshes were created, one with a 6 mm distance between struts; the other with an 8 mm distance between struts. For both types of meshes, the thickness of the PLA struts was 0.8 mm.

### Scanning Electron microscopy (SEM) imaging of reinforced scaffolds

Scaffolds were sputter coated with gold and imaged at the SEM Laboratory at the University of Alabama at Birmingham. SEM images were taken using a FEI FEG 650 SEM (Thermo) with an accelerating voltage of 10 kV in SE mode.

### Mechanical testing of scaffolds

Tensile testing was performed on three groups of scaffolds (*n* = 7 scaffolds per group): (1) electrospun scaffolds without reinforcement (control); (2) electrospun scaffolds with the 6 mm reinforcement; and (3) electrospun scaffolds with the 8 mm reinforcement. Scaffolds were cut into a dog-bone shape. Sample gauge length, width, and thickness were measured using calipers (Fisher Scientific, Hampton, NH). Measurements were taken at 3 separate locations to ensure uniformity. The sample width (13.00 + 3.0 mm) and thickness (1.00 + 0.25 mm) measurements of the specimens were used to calculate the cross-sectional area of each scaffold, A. The gauge length, L_0_, taken as the length of the reduced section of the dogbone, was measured by caliper to be 80.00 + 1.00 mm. Samples were mounted into an MTS 858 MiniBionix (Eden Prairie, MN), and then subjected to a constant displacement rate of 0.5 mm/min until the samples failed. A 100 N load cell was used to measure the force, P, while actuator displacement was recorded as the change in length, ΔL. Engineering stress was calculated as σ = P/A, while engineering strain was determined as ΔL/L_0_. Mechanical properties of tensile strength, strain at failure, and elastic modulus, calculated as the slope of the linear portion of the engineering stress-strain curve, were reported as a mean value + standard deviation.

### Statistical analysis of mechanical properties

A Shapiro-Wilk test was performed which demonstrated that the data (strength, modulus, strain at failure) were normally distributed. Once it was determined that a normal distribution was followed, analysis of variance (ANOVA) was performed for the three groups (control, 6 mm, 8 mm) for each of the mechanical properties using Excel. The results from the ANOVA showed that there were significant differences between the three groups. Post hoc tests were performed in Excel and the two-tailed *p*-value was compared to a Bonferroni corrected *p*-value. *p* < 0.05 was accepted as statistically significant.

### Implantation of reinforced scaffolds into rat cranial defects

Critical size (8 mm diameter) calvarial defects were created in Sprague-Dawley rats (*n* = 4 rats, one defect per rat). The defects were then filled with 50 mg of Bio-Oss® Anorganic Bovine Bone (ABB) bone chips (Geistlich, Princeton, NJ). Each graft site was covered with a 9 mm diameter PCL/gelatin electrospun scaffold containing the 6 mm reinforced mesh material. The implanted materials and surrounding tissues were retrieved at 20 weeks following implantation. Tissues were fixed in formalin, de-calcified and then paraffin-embedded. Tissue sections were stained with Hematoxylin and Eosin (H&E) and imaged using a Nikon Eclipse 80i microscope (Tokyo, Japan). Tissues were evaluated for potential immune cell infiltration, fibrosis, and/or other evidence of foreign body response.

## Results

### SEM imaging of reinforced electrospun scaffolds

**Fig. 1:**
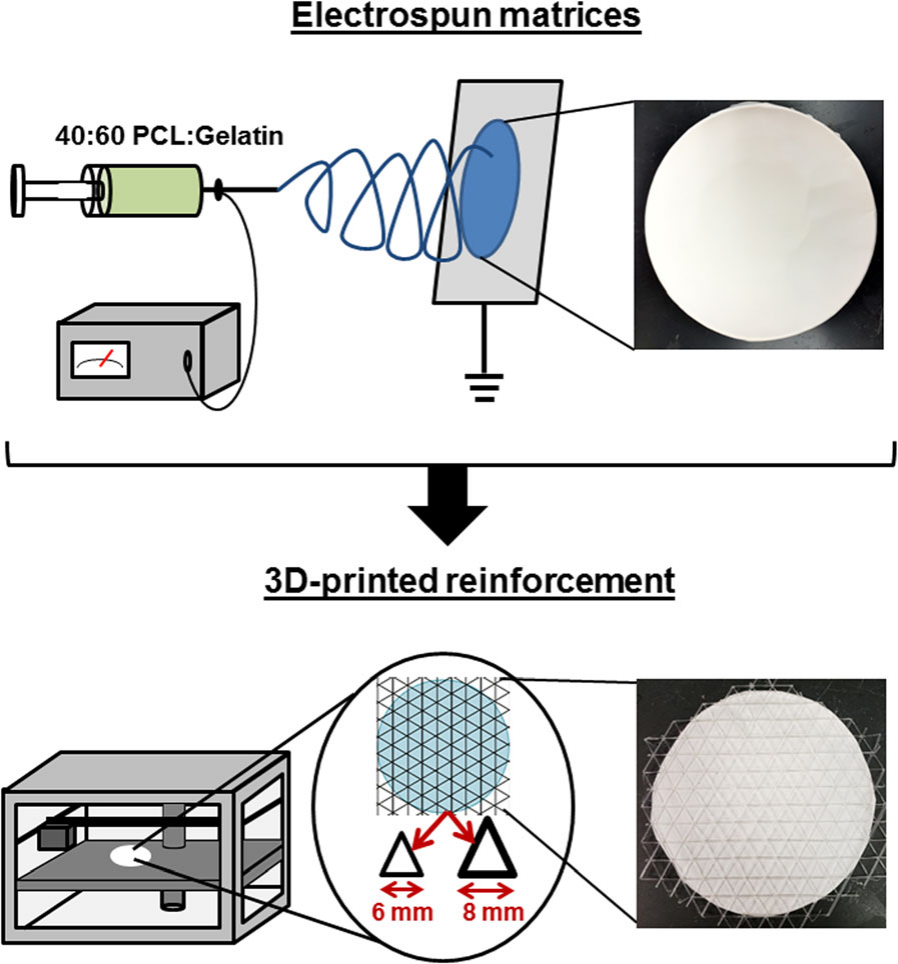
Fabrication of reinforced electrospun scaffolds. Electrospun scaffolds were produced from a 40:60 ratio of PCL:gelatin. The scaffolds were then placed in a 3D-printer and a PLA mesh was deposited onto one side of the scaffold. Two types of 3D-printed meshes were generated, one with a 6 mm distance between PLA struts, and the other with an 8 mm distance between struts

**Fig. 2:**
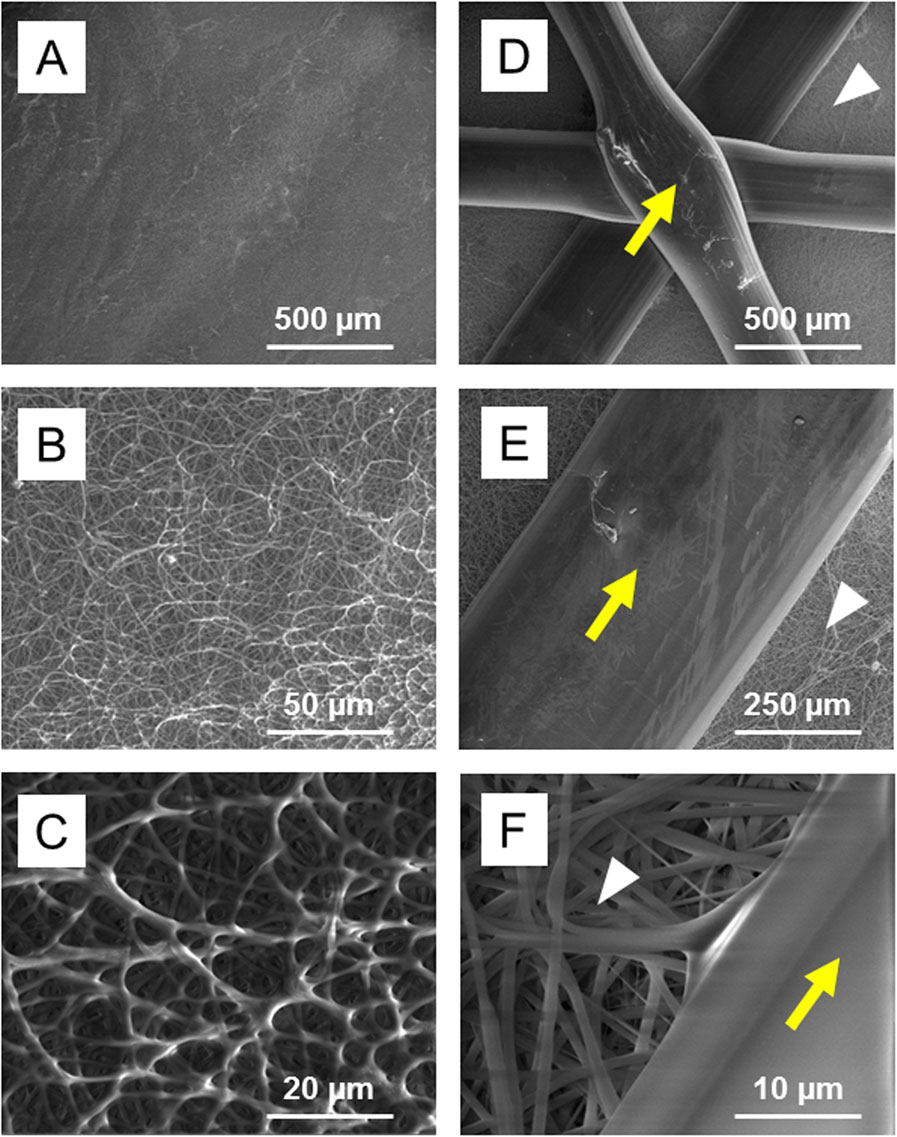
SEM imaging of reinforced electrospun scaffolds. **a**-**c** SEM images of the electrospun side of the reinforced scaffolds. Images show a uniform distribution of randomly oriented fibers. **d**-**f** SEM images of the 3D-printed side of the scaffolds. High magnification images (**f**) show that there is minimal damage to the electrospun fibers in the immediate vicinity of the 3D-printed PLA mesh. Yellow arrows depict the 3D-printed PLA. White arrowheads depict the PCL:gelatin scaffold

As a strategy for improving the mechanical properties of electrospun substrates, 3D-printing was used to deposit a PLA mesh reinforcement on one side of PCL/gelatin scaffolds (schematic diagram in Fig. 1). Two types of PLA reinforcements were generated, one with a 6 mm distance between struts; the other with an 8 mm distance between struts. Representative SEM images of the electrospun side of the scaffolds depict a uniform distribution of interconnected woven fibers (Fig. 2, panels A-C). The electrospun fiber diameters ranged from 0.5–2 μm, and the pore size diameters from 1 to 50 μm. The 3D-printed side of the electrospun scaffolds is shown in Fig. 2, panels D-F. When visualized under high magnification (Fig. 2f), it is apparent that deposition of the 3D printed layer did not compromise the woven structure of the electrospun scaffold. The electrospun fibers proximal to the PLA mesh were not melted from the heat of the 3D printing process.

### Mechanical properties of the electrospun scaffolds are enhanced by the 3D-printed PLA mesh

**Fig. 3:**
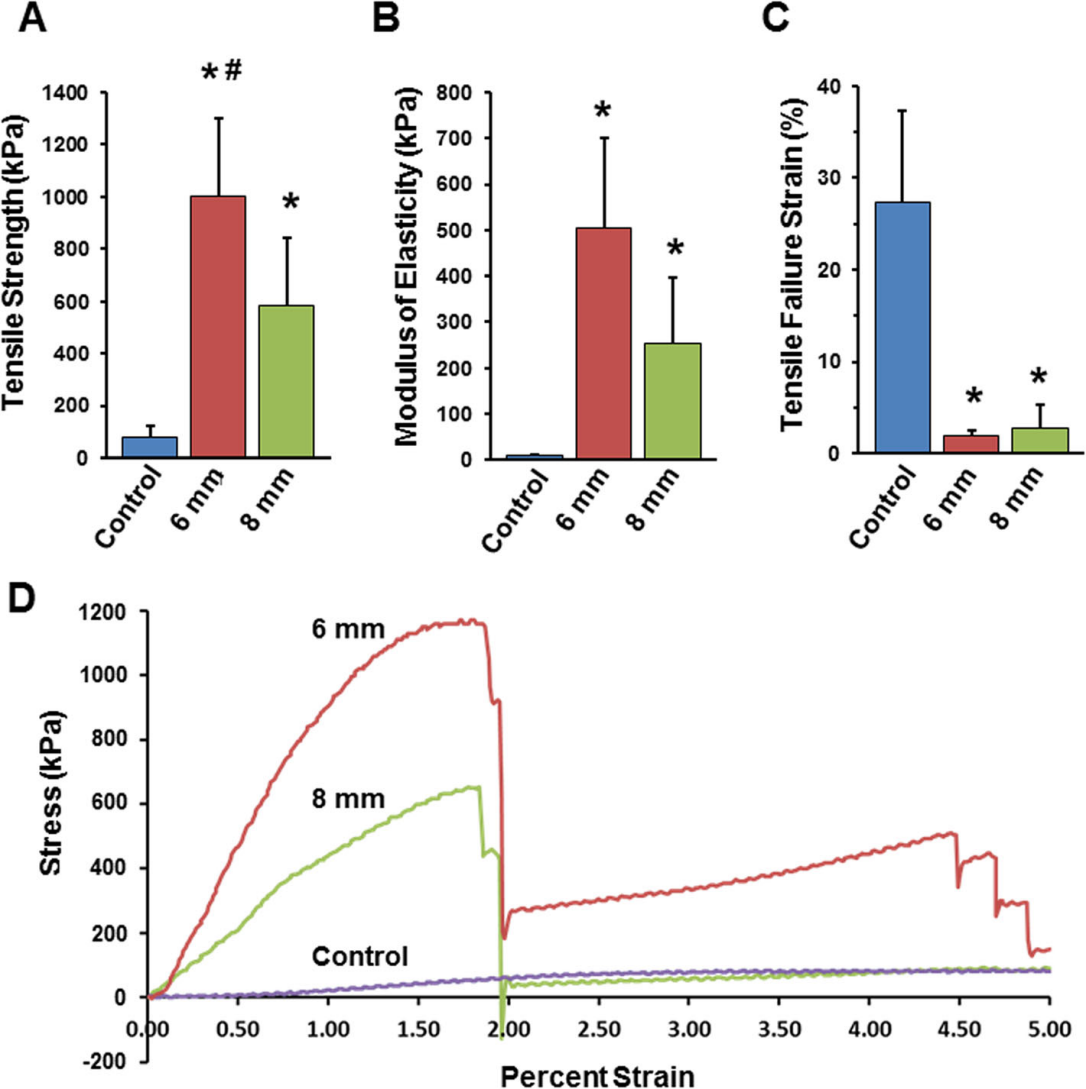
Mechanical testing confirms greater tensile strength of reinforced scaffolds as compared with unmodified electrospun scaffolds. Load to fail testing was performed on unmodified electrospun scaffolds (control) or scaffolds with either the 6 mm or 8 mm 3D-printed mesh reinforcement (*n* = 7 scaffolds per group). Scaffolds were evaluated for: **a** tensile strength, **b** elastic modulus, and **c** tensile strain. In comparison with unmodified scaffolds, the reinforced scaffolds exhibited enhanced overall strength and rigidity. **d** Representative plot of stress vs. strain. * denotes *p* < 0.05 relative to control. # denotes *p* < 0.05 relative to the 8 mm reinforced scaffold

To assess whether the mesh reinforcements enhanced scaffold mechanical properties, tensile testing was performed. Using an MTS mechanical testing system, each scaffold, under wetted conditions, was stretched using a constant linear displacement rate until mechanical failure of the scaffold was observed, indicted by a sharp drop in force. As shown in Fig. 3a, scaffolds with the 6 mm or 8 mm 3D printed mesh reinforcements had greater tensile strength than scaffolds lacking a 3D-printed reinforcement (control). Electrospun scaffolds with the 6 mm mesh exhibited the highest tensile strength, 1001 + 302 kPa. Electrospun scaffolds with the 8 mm mesh had a tensile strength of 583 + 261 kPa, and control scaffolds had a strength of 77 + 44 kPa. The higher tensile strength of the 6 mm mesh compared to the 8 mm mesh was expected, in view of the increased number of struts per area found in the 6 mm mesh. When compared to unmodified scaffolds, the 6 mm mesh scaffolds had a ~ 13 fold increase in tensile strength. ANOVA confirmed that the strength between each group is significantly different and the post hoc analyses with Bonferoni correction revealed significant differences between the control and 6 mm groups (*p* = 0.00007), the control and 8 mm groups (*p* = 0.0012), and the 6 mm and 8 mm groups (*p* = 0.0105).

The modulus of elasticity, which reflects the material stiffness, was observed to be significantly different among the groups (Fig. 3b). The moduli for the 6 mm and 8 mm scaffolds were 501 + 197 kPa and 250 + 143 kPa, respectively, whereas unmodified scaffolds displayed a much lower modulus of 8 + 4 kPa. Paired comparisons revealed significant differences between the control and 6 mm groups (*p* = 0.0002), the control and 8 mm groups (*p* = 0.0017), and the 6 mm and 8 mm groups (*p* = 0.0126).

Measurements of the percent strain at failure (Fig. 3c) similarly indicated that the 3D printed mesh markedly reduced the ductility of the scaffolds. The percent strain at failure for unmodified scaffolds was 27.35 + 9.900%, whereas values for the reinforced scaffolds were strikingly lower, specifically, 1.960 + 0.504% for the 6 mm scaffolds, and 2.779 + 2.595% for the 8 mm scaffolds. The high ductility of the unmodified scaffold during tensile testing (Fig. 3d) was anticipated, as this is a known characteristic of electrospun scaffolds [16, 18]. In this case, the control and 6 mm groups were significantly different (*p* = 0.0007) as were the control and 8 mm groups (*p* = 0.0007); however, the 6 mm and 8 mm groups were similar (*p* = 0.2912).

### In vivo compatibility of reinforced electrospun scaffolds

**Fig. 4:**
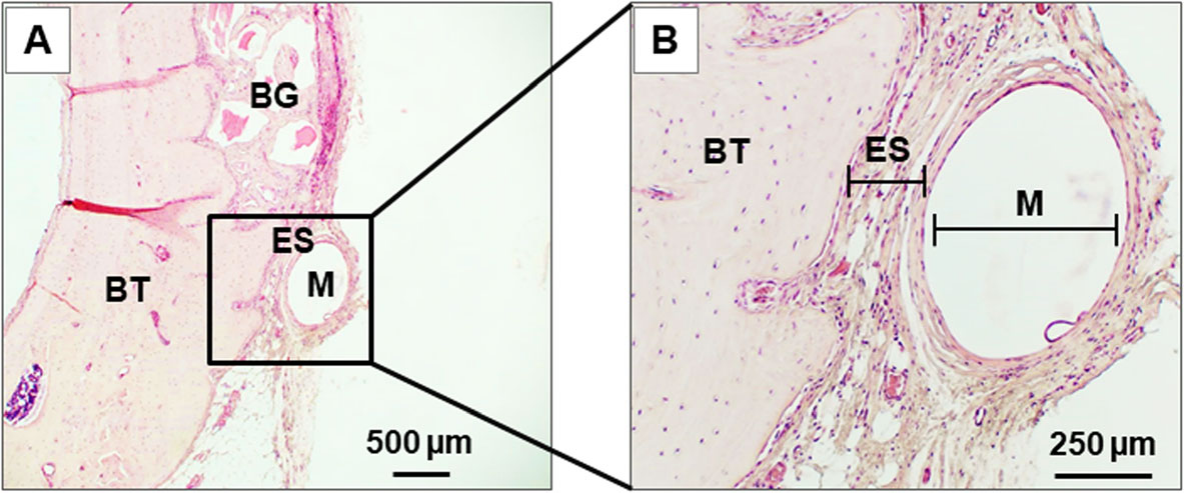
Implantation of reinforced scaffolds into rat calvarial defects. Electrospun scaffolds with the 6 mm reinforcement were used as a membrane barrier to model a bone graft surgery. Critical size defects were created in rat calvariae, and then defects were packed with ABB bone chips. The graft site was covered with the reinforced electrospun scaffold. After 20 weeks, the tissues within and surrounding the graft site were excised, formalin-fixed, de-calcified and paraffin-embedded. Tissue sections were stained by H&E (*n* = 4 rats). **a** Representative image showing that the scaffold did not elicit any immune or foreign body response. **b** Higher magnification image depicts a scaffold in direct contact with bone. M = 3D-printed PLA mesh; ES = electrospun scaffold; BT = bone tissue; BG = ABB bone graft particles

To assess biocompatibility, scaffolds were evaluated in an in vivo bone graft model. Critical size defects were created in rat calvariae, and then the defects were packed with bone graft particles. Electrospun scaffolds with the 6 mm PLA mesh were overlaid onto the defect with the 3D printed surface oriented toward the external part of the skull. After 20 weeks of implantation, the bone/implant interface was examined histologically. As shown in Fig. 4a, scaffolds did not elicit any appreciable immune or foreign body response. A higher magnification image focused on the scaffold/tissue interface (Fig. 4b) depicts the scaffold material in direct contact with bone. Although further in vivo studies will be needed, these data suggest that the reinforced electrospun scaffolds are biocompatible.

## Discussion

Electrospun scaffolds offer many advantageous features such as a fibrous matrix with tunable porosity and degradability, and high-scalability. The porous, fiber-like network of electrospun scaffolds facilitates cell attachment and infiltration into the scaffolds, processes that, in turn, promote tissue repair [18]. However, a major limitation of electrospun scaffolds is their low tensile strength [26, 27]. To address this gap, we developed a protocol for reinforcing scaffolds with a 3D-printed mesh, and showed that deposition of this mesh did not interfere with the favorable architecture of the electrospun material. More importantly, the 3D-printed PLA reinforcements significantly enhanced the tensile strength and stiffness of the scaffolds, thereby expanding the range of clinical applications for which electrospun scaffolds may be suitable. All of the components of the scaffold, gelatin, PCL, and PLA, are biodegradable, and the biocompatibility of these individual materials is well-established. Consistent with these findings, scaffolds with the 3D-printed reinforcements did not elicit any apparent immune or foreign body response upon implantation into bone defects.

Given the importance of scaffold mechanical properties in tissue regeneration, many investigators have focused on modifying the electrospinning process to enhance scaffold strength. As noted previously, blending native ECM-derived proteins with synthetic polymers such as PCL or PLA is a common approach [24, 28–30]. Fibers generated from synthetic polymers have been shown to strengthen the overall scaffold, while the naturally-derived fibers maintain their cell biocompatibility [30]. In other studies, scaffold strength was improved by electrospinning substrates with aligned fibers [31, 32]. Moffat et al. reported that aligned poly (lactic-co-glycolic acid) (PLGA) electrospun fibers exhibited a 3-fold increase in the yield strength compared with unaligned fibers [31]. Finally, metal additives have been incorporated into electrospun scaffolds [33, 34]. These additives contributed to a smaller scaffold fiber diameter, leading to increased scaffold porosity, interconnectivity and overall mechanical rigidity [33].

As an alternative to modifying the electrospinning protocol, the current study used 3D-printing to deposit a mesh reinforcement on the electrospun layer. Other investigators have similarly combined electrospinning and 3D-printing to increase scaffold strength [35–37]. As an example, Lee et al. fortified chitosan-PCL scaffolds with a 3D-printed PCL mesh [35]. The 3D-printed exoskeleton enhanced the strength of scaffolds by several fold. However, in this study, there was a lack of integration between the 3D-printed and electrospun materials due to differences in material hydrophilicity. In contrast, the scaffolds produced in the present study did not show a boundary separation between the electrospun and 3D-printed layers. The mesh reinforcement was bonded to the electrospun scaffold, but did not disrupt the fibrous architecture of this layer. This feature may have been a contributing factor to the markedly increased tensile strength of the reinforced scaffolds as compared with unmodified electrospun scaffolds.

## Conclusions

3D printed PLA mesh reinforcements significantly increase the strength and stiffness of electrospun scaffolds, and reduce scaffold ductility, without compromising the ECM-like architecture of the electrospun material, or adversely affecting its biocompatibility. The addition of the 3D printed mesh is technically straightforward and can be applied to any type of electrospun scaffold, highlighting adaptability of this approach to scaffolds of varying biochemical composition or structure. Furthermore, the differences in the mechanical propoerties imparted by the 6 mm vs. 8 mm reinforcements point to a potential strategy for tuning the strength and stiffness of electrospun scaffolds through the use of meshes with different sizes and shapes. In sum, the current investigation suggests that the mechanical properties of electrospun scaffolds can be markedly improved by the addition of tunable 3D printed meshes, while preserving the desirable aspects of the electrospun material.

## Data Availability

Data sharing not applicable to this article as no datasets were generated or analysed during the current study.

## Abbreviations

ABB: Anorganic bovine bone
ANOVA: Analysis of variance
ECM: Extracellular matrix
FDA: US Food and Drug Administration
H&E: Hematoxylin and eosin
HFP: Hexafluoropropanol
PCL: Poly(ε-caprolactone)
PHBV: Poly (3-hydroxybutyrate-co-3-hydroxyvalerate)
PLA: Poly (lactic acid)
PLGA: Poly (lactic-co-glycolic acid)
PVD: Poly (vinylidene fluoride)
SEM: Scanning electron microscopy
